# Retraction: Deep multi-modal intermediate fusion of clinical record and time series data in mortality prediction

**DOI:** 10.3389/fmolb.2025.1640945

**Published:** 2025-06-13

**Authors:** Frontiers Editorial Office

The journal retracts the 08 March 2023 article cited above.

Following concerns regarding the originality of the article, an investigation was conducted in accordance with Frontiers’ policies. It was found that the complaint was valid and that the article should be retracted, because of an unacceptable level of similarity without meaningful or appropriate attribution to an article published by Deznabi, I., Iyyer, M., and Fiterau, M. (2021). “Predicting in-hospital mortality by combining clinical notes with time-series data,” in *Findings of the Association for Computational Linguistics*: *ACL-IJCNLP 2021*, 4026–4031. doi: 10.18653/v1/2021.findings-acl.352.

This retraction was approved by the Chief Editors of Frontiers in Molecular Biosciences and the Chief Executive Editor of Frontiers. The authors have agreed with the concerns regarding the failure to attribute adequate credit to the article published by Deznabi et al. The authors have not agreed with the remaining concerns raised.

